# Mechanochemical and Size Reduction Machines for Biorefining

**DOI:** 10.3390/molecules25225345

**Published:** 2020-11-16

**Authors:** Igor Lomovskiy, Aleksey Bychkov, Oleg Lomovsky, Tatiana Skripkina

**Affiliations:** Institute of Solid State Chemistry and Mechanochemistry SB RAS, Kutateladze Str. 18, 630090 Novosibirsk, Russia; lomovsky@solid.nsc.ru (I.L.); bychkov_a@solid.nsc.ru (A.B.); lomov@solid.nsc.ru (O.L.)

**Keywords:** mechanochemistry, energy consumption, biorefining, plant raw materials, mechanical pretreatment, scaling

## Abstract

In recent years, we have witnessed an increasing interest in the application of mechanochemical methods for processing materials in biomass refining techniques. Grinding and mechanical pretreatment are very popular methods utilized to enhance the reactivity of polymers and plant raw materials; however, the choice of devices and their modes of action is often performed through trial and error. An inadequate choice of equipment often results in inefficient grinding, low reactivity of the product, excess energy expenditure, and significant wear of the equipment. In the present review, modern equipment employing various types of mechanical impacts, which show the highest promise for mechanochemical pretreatment of plant raw materials, is examined and compared—disc mills, attritors and bead mills, ball mills, planetary mills, vibration and vibrocentrifugal mills, roller and centrifugal roller mills, extruders, hammer mills, knife mills, pin mills, disintegrators, and jet mills. The properly chosen type of mechanochemical activation (and equipment) allows an energetically and economically sound enhancement of the reactivity of solid-phase polymers by increasing the effective surface area accessible to reagents, reducing the amount of crystalline regions and the diffusion coefficient, disordering the supramolecular structure of the material, and mechanochemically reacting with the target substances.

## 1. Introduction

In recent years, there has been an increasing interest in the application of mechanochemical methods for processing materials in biomass refining techniques [1,2]. These methods have gained an especially strong position in spheres such as the fabrication of drugs, dietary supplements, components of functional foods, and sport nutrition products containing thermally unstable substances of a polyphenolic and protein nature (antioxidants, enzymes, vitamins, and some probiotics) [3,4,5,6,7]. The term “mechanochemical” means that treatment results not only in grinding and other mechanical action, but also chemical reactions [8]. Mechanochemistry is a branch of chemistry that studies changes in the properties of substances and their mixtures, as well as physicochemical transformations occurring as a result of mechanical influence (during processing or immediately after it). There are traditionally strong mechanochemical research centers in Germany, USA, China, Japan, Russia, and EU countries. The key manufacturers of mechanochemical equipment are located in the same countries: RETSCH (Haan, Germany), FRITSCH (Idar-Oberstein, Germany), NETZSCH-Feinmahltechnik GmbH (Selb, Germany), NOVIC (Novosibirsk, Russia), Activator Corporation (Novosibirsk, Russia), Weifang Zhengyuan Powder Engineering Equipment Co. (Weifang, Shandong, China), Ningguo Weida Casting Co (Ningguo, Anhui, China), MAKINO Corporation (Tokoname-shi, Aichi, Japan), U.S. Stoneware (East Palestine, OH, USA), Union Process (Akron, OH, USA), and Desintegraator Tootmise (Tallinn, Estonia).

From the perspective of its structure and physicochemical properties, the biomass and the processes occurring upon biorefining are objects of an interdisciplinary research in polymer chemistry, solid state chemistry, chemical engineering, and material sciences [9,10]. A sufficient foundation in this area was created in the 1930–1970s; however, most of the studies focused on the conventional liquid-phase manufacturing processes (extraction, recrystallization, flotation, etc.) [11]. Publications devoted to solid-phase mechanochemical processes addressed the questions regarding aging of polymers and resins utilized in manufacturing procedures [12,13].

Since the world has been shifting towards the environmentally friendly principles of green chemistry, in recent years there has been renewed interest in studying the mechanisms and the kinetics of solvent-free processes occurring upon exposure of polymers to mechanical impact [14,15,16,17]. The published reviews [18,19,20,21] devoted to the foundations of mechanochemical processes infer that the mechanochemical approaches have become a global trend. However, there is not enough fundamental data to put the technologies being developed into common practice. Thus, in their review, G. Cagnetta et al. [22] evaluated the economically and energetically sound mechanochemical biodegradation of polycyclic aromatic hydrocarbons and found that it is feasible only in large high-energy mills (with production capacity of several tons/h), which are not very common on the market.

Some assessments demonstrated that the costs of grinding equipment in the mineral extraction industry can be as high as 60% of the total capital investment, while energy and maintenance expenses can amount to 40% of net cost of the final product [23]. However, the attempts to extrapolate the experience gained from inorganic materials and minerals to the systems consisting of polymers usually fail. The micromechanical activation processes involved in mechanochemical pretreatment of organic (and, in particular, polymeric) materials often differ from those typical of inorganic materials; furthermore, phase transitions occur via different mechanisms, while the mobility, reactivity, and stability of molecules also differ [24,25,26].

Because of the insufficient knowledge regarding the principles of action of the modern mechanochemical equipment, the mechanochemical stages during the refining of biomass are often performed through trial and error. Again and again, the researchers’ own experience makes them draw an obvious conclusion—that the energy required for pretreating material until the same parameter (size, specific surface area, or reactivity) is achieved can differ several times depending on what equipment was chosen [27,28,29].

This approach views the mechanochemical equipment as a “black box” and does not allow one to promptly modify the manufacturing processes to organize integrated waste-free refining of the entire range of plant raw materials, use hardly accessible and nonreactive substances in biorefining, increase the yield and selectivity of the processes, and speed up heterogeneous reactions.

The objective of this study was to examine and compare modern equipment employing various types of mechanical impact, which show the highest promise for mechanochemical pretreatment of plant raw materials.

Currently, there are many models of solid body destruction [30]. However, all of them do not pay proper attention to the nuances of interactions between the material being milled and grinding bodies; neither do they describe the processes taking place upon mechanical pretreatment of large quantities of particles of the material being milled [31]. Therefore, a simple classification based on the type of mechanical impact (crushing, cleavage, cutting, sawing, abrasion, constrained and free impact) applied to the particles is used when discussing mills and mechanochemical activators. It is clear that most grinders employ several mechanisms of impact. Therefore, the impact making the maximum contribution is usually implied when characterizing a certain type of equipment.

In order to perform cleavage, cracking and sawing, the particle size needs to be comparable to the size of the working bodies of a grinder. These types of impact can be used only for primary pretreatment of the feedstock whose particles are larger than several centimeters. Crushing is only appropriate for brittle materials, while plant raw material is not brittle. In this connection, the equipment employing cutting, abrasion, and impact (both free and constrained) is utilized for grinding plant raw material.

## 2. Mechanochemical Equipments for Biorefining

### 2.1. Disc Mills

**Figure 1:**
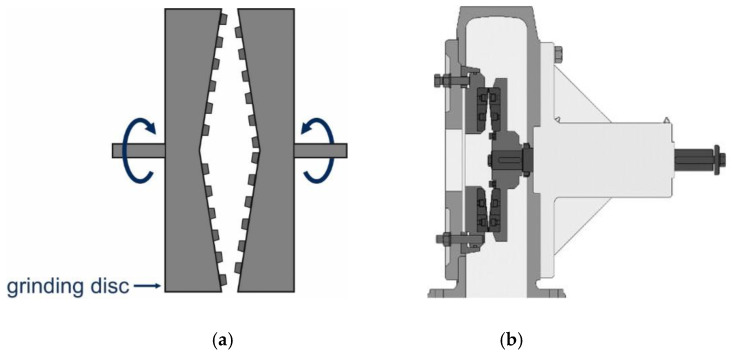
Schematic showing the principle of action (**a**) and schematic representation of a disc mill (**b**). Reproduced with permission from Roland Nied, Handbook of Powder Technology; published by Elsevier, 2007 [32].

Disc mills are probably the most ancient type of grinding equipment. Their principle of action is as follows: the material passes between two rotating discs, causing friction of particles against the discs and grinding (Figure 1a). Mills with a variable gap between the discs and discs with notches are used to increase the milling efficiency (Figure 1b). Many types of notch shapes for discs are currently known, but there is still no general opinion for which type is the “correct” one.

Along with roller mills, disc mills are the main types of grinders used for milling grains of wheat, maize, and other crops. Product grain size typically ranges from 300 to 200 µm. Disc mills have also proved to be efficient in milling fibrous materials (e.g., during pulp preparation in pulp and paper industry) [33,34,35].

Disc mills compare favorably to their analogs as they have a narrow particle size distribution, which depends on the gap between the discs. Their main shortcoming is that they have a small operating volume since the feedstock is pretreated in the thin gap between the discs. Because of this fact, it is impossible to design disc mills with a production capacity of over 500 kg/h. Energy consumption during the milling of wood material to a level of several dozen micrometers is estimated at 4.2 MW·h/ton [36], being several times higher than energy consumption of other mills. It is unreasonable to increase disc rotation speed or radius to enhance milling efficiency. These actions will elevate shear strain and cause a temperature jump in the pretreatment zone, making the target substances decompose.

### 2.2. Attritors and Bead Mills

**Figure 2:**
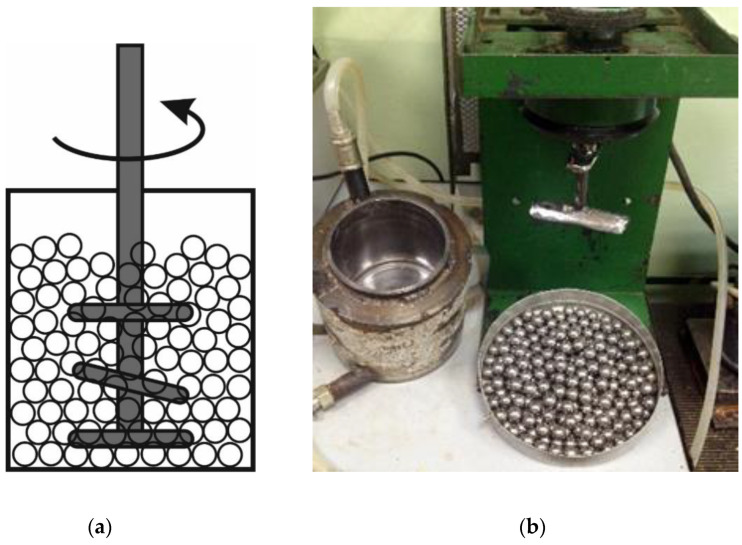
Schematic showing the principle of action (**a**) and a real-world example of an attritor designed at the Institute of Solid State Chemistry and Mechanochemistry, SB RAS (**b**).

Attritors refer to the kind of equipment composed of a reactor that is almost completely filled with grinding bodies and has an impeller drowned in them (Figure 2). As the impeller is rotating, it carries the grinding bodies with it. The moving grinding bodies grind the raw material between them.

The major advantage of this type of equipment is that the particles being ground cannot leave the contact zone between the grinding balls. When the size of the grinding media is sufficiently small (steel or ceramic beads ≤300 µm in diameter), nanosized particles can be obtained. The technological feasibility of obtaining grinding bodies several hundred micrometers in size has allowed one to achieve a breakthrough in fabrication of modern ultrafine pigments for laser printers and dyes.

The major shortcoming of attritors is that it is difficult to isolate the grinding product from the grinding media. Today, this is possible only when treatment is conducted in the presence of liquid whose flow carries the material inside the grinding zone [37]. Industrial-scale attritors are extremely rarely used for dry grinding. Furthermore, this type of equipment is characterized by high wear of grinding media during treatment, which increases the cost of equipment operation and causes product contamination.

### 2.3. Ball Mills

**Figure 3:**
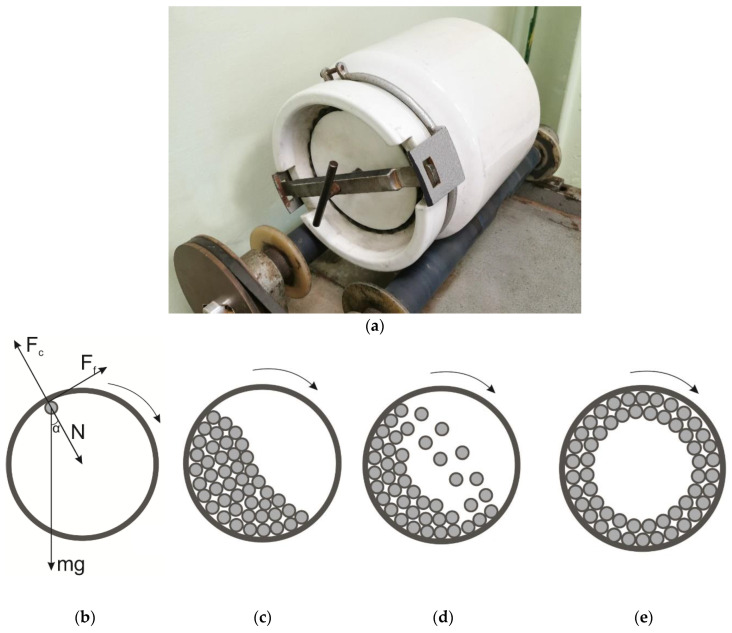
A real-world example (**a**), the effect of forces (**b**) and the types of motion of grinding bodies in a ball mill: (**c**)—rolling over; (**d**)—falling; (**e**)—rolling.

A ball mill is one of the simplest and most common types of mechanochemical equipment (Figure 3) [38,39]. Sometimes this type of equipment is referred to as a “roller mill” or a “roller crushing mill” by mistake [40], because the mill drive resembles rotating rollers. In many mechanochemical studies, this mill is used as a standard.

The principle of action of a ball mill is as follows. A rather large number of grinding bodies and the material being ground are placed inside a cylinder rotating around its central axis. Due to forces of friction between the cylinder walls and the milling bodies, the bodies start to move and grind the raw material. Sometimes grinding bodies are not used, and particles of the material grind themselves. This process is known as “auto-grinding”.

The main type of mechanical impact in a mill depends on rotational speed (Figure 3c–e). At small rotational speeds, the grinding bodies roll over, and abrasion is the main mechanism of mechanical action (Figure 3c). As the rotational speed is increased, the grinding bodies are detached from the wall in the highest point of trajectory and fall, thus ensuring impact effects (Figure 3d). As the rotational speed is increased further, the grinding bodies become distributed along the walls and roll, making the raw material undergo abrasion again but with a lower efficiency (Figure 3e) [41]. The boundary condition for the rolling mode (Figure 3e) is rotational speed n = 42∙D^−1/2^ (rpm), where D is the diameter of a grinding chamber (m) [42]. From the viewpoint of energy, the most efficient mode is the one with falling bodies. Therefore, the rotational speed equal to 65–80% of the critical one is used for the falling mode. The critical rotational speed is calculated for a grinding body in the upper point of the grinding chamber by assuming that the centrifugal force is equal to gravity acting on the grinding body. The effect of force F_f_ (Figure 3b), which indicates the cohesion between the grinding body and the inner wall of the chamber, is not taken into account. When the grinding body has not yet reached the top the top point (Figure 3b), the boundary condition for its detachment from the wall and it starting to fall can be written as:

mg + Ncosα = F_c_cosα + F_f_sinα (1)

where N is the support reaction force that is maximal when the grinding body passes the bottom point of the reactor and minimal in its top point. F_c_ is the centrifugal force.

Therefore, one can see that cohesion contributes to the final mode where the grinding bodies are rolled over, so many manufactured industrial-scale ball mills have a rough surface or even are equipped with special grooves on their inner surface [43]. These grooves can lift the grinding bodies to the required height and ensure the impact mode of grinding. However, this approach is efficient mainly for grinding brittle inorganic materials. In the case of fibrous plant feedstock or synthetic polymers, it is better to choose activator mills with smooth inner walls and conduct treatment in the rolling-over mode in which the attrition action prevails.

The size, quantity, and shape of grinding bodies also affect the efficiency of mechanochemical processes occurring in a ball mill; energy consumption during milling largely depends on optimization of these parameters. Grinders of all types are well-suited to laboratory-scale research. Even if treatment conditions are not efficient, the material will still be ground, although it might take several dozen or even hundreds of hours to achieve the desired results. In the general case, it is recommended to use steel balls twice as large as the initial size of the raw material particles as grinding bodies. In order to minimize ineffective collisions, the volumes of the loaded grinding media and raw material need to be approximately 30% of the total mill charge volume [44].

This type of grinder has a number of drawbacks preventing its use for treating plant raw material. The first drawback is a low production capacity compared to that of other grinding equipment. This problem can be partially solved by scaling-up the geometric size of ball mills. Thus, parameters of a ball mill specified by manufacturers are usually as follows: maximum diameter, ~5 m; maximum length, 15 m; energy consumption, up to 4 MW; a production capacity, with respect to sand, of 120 tons/h. For plant raw material (whose packed density significantly differing from that of sand so the time required for milling is also different), the production capacity of the same mill will be no higher than 5–15 tons/h. Thus, energy consumption during milling of wood material can be estimated at 0.4 MW·h/ton. The second factor making it difficult to use this equipment is that a ball mill generates high-frequency mechanical vibrations oriented vertically downward [45]. Therefore, this type of equipment needs to be mounted on a separate foundation not connected to the rest of the building structure.

### 2.4. Planetary Mills

**Figure 4:**
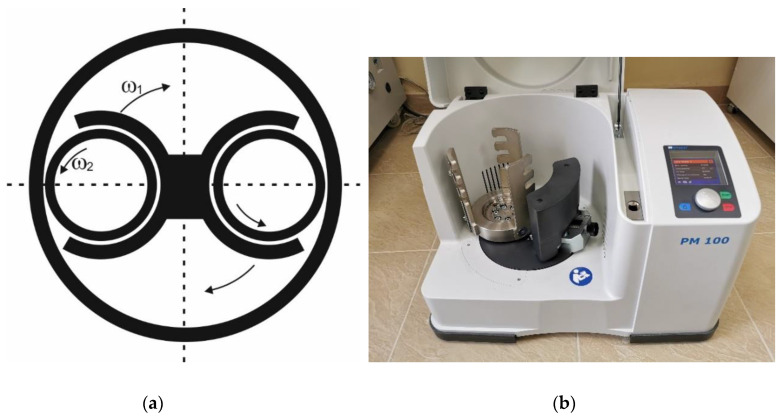
A planetary mill: (**a**) the flow diagram of a planetary mill; (**b**) photograph of the real-world equipment manufactured by Retsch GmbH. The photo was made by Skripkina T.S, 2020.

Planetary activator mills are a specific development of the concept of ball mills. In this type of equipment, the reservoirs for grinding are secured to a frame and rotate around a common axis. Meanwhile, the reactors rotate around their axes in a direction opposite to that of frame rotation (Figure 4).

Disintegration occurs due to interactions between the raw material and grinding bodies, which are put into motion by the centrifugal force, the Coriolis force, and gravity. There is no critical difference between the motion of grinding bodies in a planetary mill and in a ball mill, which has a simpler design [46].

To provide characteristics of the equipment and compare similar grinders from different manufacturers, planetary mills are characterized by using a special parameter: acceleration of a milling body at an instant when it is detached from the wall. Planetary rotation allows accelerating the grinding bodies to 2000 m/s^2^ and even more [47], but the grinding modes providing acceleration of 200–600 m/s^2^ are the most common ones. Because of the high intensity of a single mechanical action and the high density of supplied energy, not only grinding but also more complex processes take place in planetary activators [48,49,50,51,52]. The development of high-intensity grinders has significantly increased the number of studies focused on chemical reactions and processes induced by mechanical loading [18,53,54,55].

An important feature of high-intensity mechanical treatments is that the local region where a grinding body interacts with the raw material is characterized by a significantly elevated temperature. When grinding bodies are accelerated to a value above 200 m/s^2^, the temperature may rise by several hundred degrees [56]. For plant raw material, such an elevation in temperature may cause decomposition of some low-molecular-weight substances, polymer carbonization, and other undesirable processes. Therefore, the milling modes with grinding body acceleration limited to 50–200 m^2^/s are usually used in mechanochemistry of organic molecules and for grinding plant raw materials.

The nonflow-type planetary mill is limited to a capacity of 3 kg/h, while the continuous-flow type mill can process up to 6 tons/h. Unfortunately, the portfolio of commercially available continuous-flow planetary mills is very limited because of the difficulties related to ensuring uniform feeding of raw materials and product removal from the treatment zone [57].

### 2.5. Vibration and Vibrocentrifugal Mills

**Figure 5:**
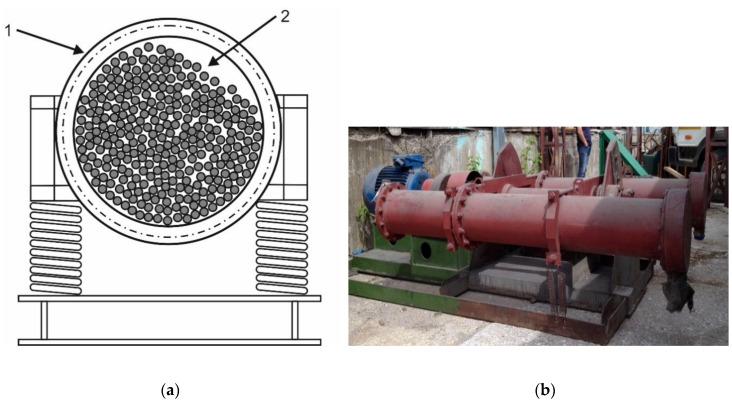
A vibration mill: (**a**) flow diagram; (**b**) photograph of the real-world K-50 equipment manufactured by NPO Novic Ltd. [60] (1—mechanochemical reactor; 2—the milling bodies); the photo was taken by Skripkina T.S, 2019.

Vibration mills are a common class of equipment used for fine and ultrafine grinding and conducting mechanochemical processes [58,59]. The fundamental difference between vibration mills and the ball ones is that instead of circular motion, the reactor makes vertical vibrations (Figure 5). Therefore, within the reference frame of the reactor, motion of the grinding bodies can be regarded as motion in the variable gravity field. Expressed as acceleration, this field can be as high as 200 m/s^2^.

The amplitude of reactor vibrations is usually no higher than 20 mm, which correspondingly limits the trajectories of the grinding bodies and allows one to increase the volume of grinding balls and raw material loaded into the mill (compared to that for ball and planetary mills). One of the varieties of vibration mills is a toroidal mill. It is a continuous-flow-type vertical vibration mill, where the vibration action on the grinding bodies is perpendicular to the Earth’s surface, which reduces the impact of gravity forces on grinding and ensures that the loss of supplied energy is minimal. The authors do not know any examples of using this type of equipment for biorefining or polymer processing.

Akin to the industrial-scale ball mills, the major limitation for industrial use of vibration grinders is that they need to be mounted on a special foundation not connected to the building foundation and whose weight is several times higher than that of the equipment.

**Figure 6:**
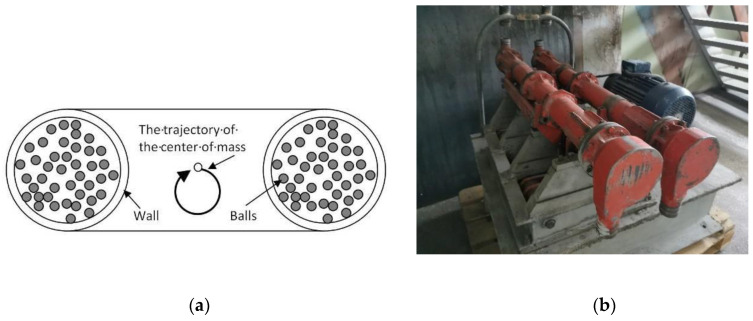
A vibrocentrifugal mill: (**a**) schematic cross-sectional view; (**b**) an example of the real-world equipment designed by the engineering department of the Institute of Solid State Chemistry and Mechanochemistry, SB RAS.

To minimize the vibration-related problems and reduce the load on the structural elements of the mills, the vibration mills have been modified; the unbalanced drive was replaced with a second reactor moving antiphase, while vertical vibrations were replaced with rotary motion. This type of equipment is known as a vibrocentrifugal mill (Figure 6).

Within the reference frame of the reactor, the motion of the grinding bodies in this equipment is identical to that in a ball mill. Vibrocentrifugal mills are considered to be the closest analog of planetary mills [61,62].

**Figure 7:**
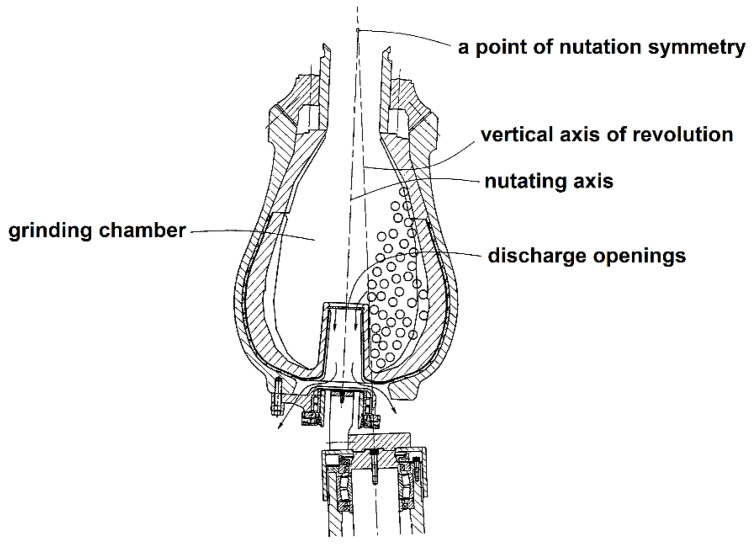
Schematic of a centrifugal nutation mill. Modified from Hoyer DI, Hills P. Centrifugal grinding mills. Patent US 7,070,134 B1, 2006.

A high energy density, which allows the acquisition of particles much smaller than those produced in a ball mill, is an advantage of this type of grinder. However, for thermally unstable organic molecules and plant feedstock components, this property causes temperature elevation in the grinding zone and undesirable oxidation, decomposition, and depolymerization processes. The grinding bodies need to have certain space to move; therefore, vibrocentrifugal mills are characterized by a large empty volume compared to that of vibration mills. Once particles of low-density feedstock (pigments, plant raw material, peat, and plastics) are ground to a certain size, they form a lightweight and mobile suspension inside the mill, which decreases the probability of collisions between the grinding bodies and feedstock particles and reduces the process efficiency. This drawback is not so noticeable for centrifugal nutation mills (nutators), where the operating volume of the mill is filled with the grinding bodies more densely and rotates along a circular path with a nutating central axis [63,64] (Figure 7).

### 2.6. Roller and Centrifugal Roller Mills

**Figure 8:**
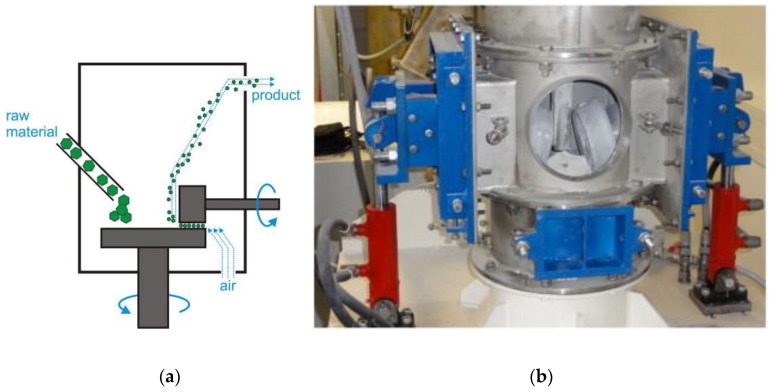
A roller mill: (**a**) schematic showing the principle of action; (**b**) an example of the real-world roller mill (VRM200), reproduced with permission from A. Boehm, P. Meissner, T. Plochberger, International Journal of Mineral Processing; published by Elsevier, 2015. [66].

Roller mills are a type of grinder in which the feedstock is simultaneously subjected to two types of impact: abrasion and crushing (Figure 8) [65].

In the first samples of the equipment, the grinding products were not removed from the mills, so grinders of this type were operating discontinuously. In modern roller mills, there is an airflow that carries fine particles away from the grinding zone. A high production capacity can be achieved due to this fact, but the particles being removed usually have a broad size distribution.

Today, ball mills are gradually giving way to roller mills in the manufacturing of cement and other dry mortars. An advantage of this type of grinder is good scalability (up to production capacity of 1000 tons/h). However, the problem associated with obtaining narrow fractions during grinding of polymers or plant raw material still needs to be solved. It should also be mentioned that it is difficult to control temperature in the grinding zone in this type of roller mill, so plant raw material gets overheated.

**Figure 9:**
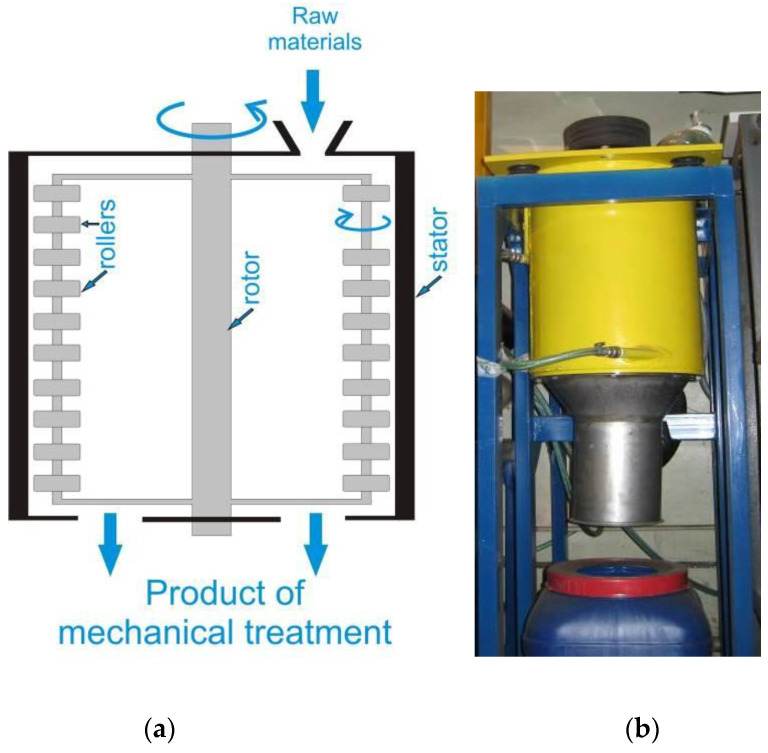
A centrifugal roller mill: (**a**) schematic; (**b**) an example of the real-world grinder designed by the engineering department of the Institute of Solid State Chemistry and Mechanochemistry, SB RAS.

Centrifugal roller mills are a modern alternative to roller mills; in them, the roller axes are oriented vertically, while the feedstock is ground as it gets between the rollers and the stator (Figure 9) [67].

This type of equipment is characterized by a narrow particle size distribution. Furthermore, it is easy to arrange stator cooling and control temperature in the grinding zone. Centrifugal roller mills with a production capacity of 50–150 kg/h have already been designed. However, engineering efforts are still ongoing, and some manufacturers declared that their equipment has a production capacity up to 5 tons/h.

### 2.7. Extruders

**Figure 10:**
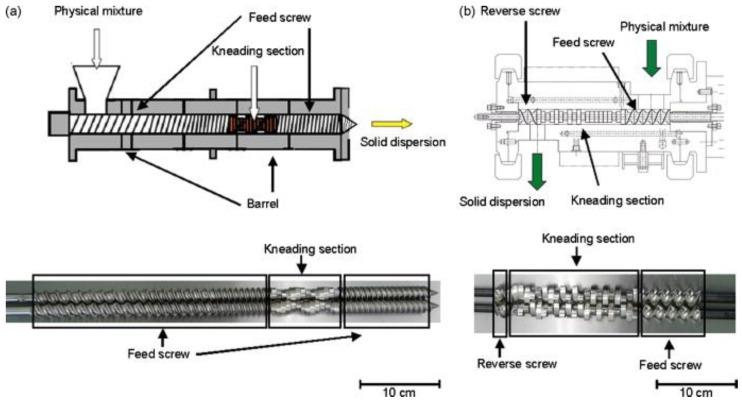
Schematic view of the (**a**) extruder and (**b**) kneader. Reproduced with permission from Y. Shibata, M. Fujii, Y.Sugamura, R. Yoshikawa, S. Fujimoto, S. Nakanishi, Y. Motosugi, N. Koizumi, M. Yamada, K. Ouchi, and Y. Watanabe, International Journal of Pharmaceutics; published by Elsevier, 2009 [68].

Extruders belong to one of the few classes of equipment in which mechanical treatment is not necessarily accompanied by grinding. A screw (usually one or two) is the main working part of the extruder (Figure 10). As the screw rotates, it captures the material and presses it along the treatment zones. At a sufficiently large volume of loaded raw material, it strongly interacts with the extruder walls due to friction during pressing, thus causing substantial plastic deformation of the raw material. At the extruder end, there is usually an orifice with a diameter chosen so that the material passing through it is also significantly deformed.

Today, extruders are extensively used for biorefining [69] and the production of homogeneous mixtures [70] and composites [52]. In 2019, International Union of Pure and Applied Chemistry (IUPAC) announced reactive extrusion to be one of the ten technologies “having a potential to make our planet more sustainable” [71].

During grinding of polymeric materials, strong plastic deformation results in significant friction at the level of macromolecular chains, causing heating of the material to several hundred degrees. If the raw material being treated contains water, it boils upon heating, and autoclaving-like conditions are generated. This fact makes designing extruders where plant raw materials can be treated significantly challenging. The pressure decreases abruptly as the material passes through the outlet orifice, making the raw material undergo steam explosion treatment.

Hence, extrusion is a combination of strong plastic deformation, short-term autoclaving, and steam explosion treatment. It is noteworthy that extrusion is one of the methods ensuring mass transfer inside solid particles, which allows one to fabricate mechanocomposites and conduct reactions.

The advantages of extrusion are as follows: it can be used to grind materials with a high water content and large particle size of the raw material (up to several dozen millimeters) [72]. One of the major drawbacks of extruders is the high mechanical load applied to screws, making it rather challenging to design the equipment that would have a production capacity more than 100 kg/h.

Hence, for the entire range of aforementioned equipment employing the principle of constrained impact of a grinding body on the raw material, there are problems reducing the probability of using this equipment for industrial-scale grinding of plant raw material, microbial biomass, polymers, peats, and thermolabile substances. It is reasonable to utilize the equipment whose action is based on constrained impact or abrasion in the cases when one needs to ensure contact between particles in the solid-phase for composite formation or occurrence of mechanochemical reactions. Roller and centrifugal roller mills are the most promising types of the equipment listed above as they are simultaneously characterized by a high production capacity and relatively low energy consumption.

In all the aforementioned mills, a feedstock particle needs to get between the grinding bodies so that grinding or a chemical reaction can take place. In the optimal mode, the probability of this event is estimated to be 50–70% of the total number of collisions, so 30–50% of energy is consumed for “vain” collisions between the grinding bodies. Therefore, if the purpose is grinding only (not accompanied by disintegration of the cell structure or occurrence of mechanochemical processes), it is more reasonable to use the equipment where grinding bodies do not interact with the walls or each other (free-impact mills).

### 2.8. Hammer Mills, Knife Mills, Pin Mills, and Disintegrators

This group of equipment does not have a common name as it involves several modifications of grinders, with minor differences in details. A shared feature of this type of equipment is that a disc (or two discs) with grinding elements secured on surface acts as a milling body. The raw material typically passes from the disc center into the treatment zone and is dispersed to the chamber periphery by centrifugal force, where it is exposed to grinding elements.

**Figure 11:**
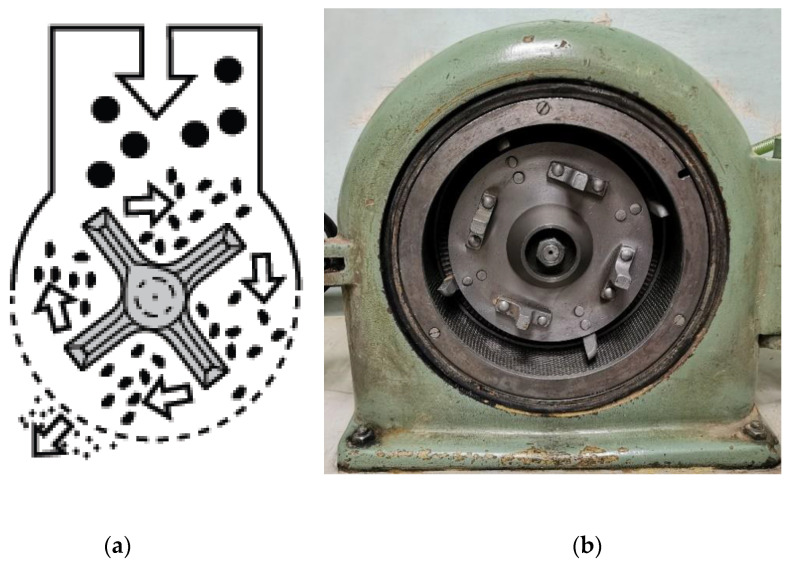
A hammer mill: (**a**) schematic of the mill; (**b**) an example of the real-world hammer mill.

Hammer and knife mills are the most common varieties [73,74]. The main difference of these mills from the other ones consists of the large size of grinding bodies (hammers and knifes, respectively) and their small number (usually four to eight per disc). Another difference is that a screen (a mesh) is mounted on the side end of the grinding zone (Figure 11). Grinding involves two stages. During the first stage, particles are ground as they collide with the milling bodies. Next, the particles are pressed through as the rotor moves through the screen, resulting in their crushing and abrasion.

This type of equipment can be characterized by a high production capacity (several dozen tons per hour) and low specific energy expenditure [75]. Due to the screen, the size distribution of product particles is much narrower than that of particles ground in any other free-impact mills.

The key factors restraining the development of hammer mills are as follows: (1) production capacity drops with decreasing mesh size and (2) screens with a mesh size less than 1 mm that can withstand high mechanical loads are very expensive [76]. In this connection, hammer mills are usually utilized as a tool for the pregrinding of raw material and reducing the particle size from a several dozen centimeters to several millimeters [77].

**Figure 12:**
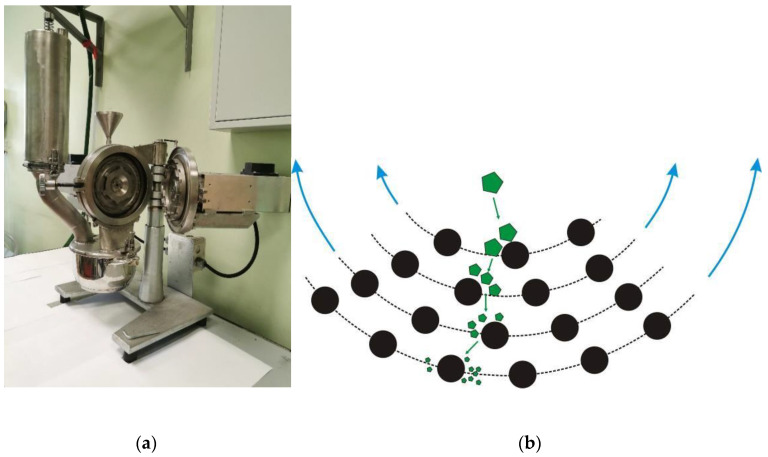
An example of the real-world disintegrator (**a**) and a schematic showing the motion of particles in the disintegrator (**b**).

Disintegrators are another type of equipment based on the rotation of discs with grinding elements; sometimes they can be called a “dismembrator” in the case when one disk is rotating and another one is stationary (Figure 12). In this type of equipment, the raw material is fed into the treatment zone from the disc center and is exposed to repeated collisions with rotor pins rapidly moving (at a speed up to 300 m/s) as it moves from the center towards the periphery.

As a particle moves, it typically undergoes two to four impact events. Because the average number of impact events is small, there is a high probability that the particle will experience two to six impact events [78]. This leads to a broad size distribution of product particles. The rotation of disintegrator rotors generates an intense airflow through the equipment, so particles are rapidly removed from the grinder, which also has an unfavorable effect on grinding quality. Therefore, disintegrators are often used together with a product classification system to return coarse particles into the grinding zone.

Several features shared by hammer mills and disintegrators are worth mentioning. First, the internal structure of plant raw materials is often retained after grinding; fibrous materials remain fibrous after grinding (of course, if particle size is larger than the size of a single fiber). This fact is used extensively for producing composite materials and wood–plastic composites [79]. Second, a certain impact strength needs to be attained to ensure efficient grinding. This can be achieved by applying a high rotational speed of discs. However, at a certain speed, a region of thickened air is formed at the butt end of the grinding elements, making fine particles fly around the grinding body rather than collide with it.

The minimal possible particle size that can be attained using this type of equipment is several times larger than that for constrained-impact mills. Thus, a particle size less than 75 µm can rarely be achieved for plant raw materials. Energy consumption of the free-impact mills is estimated at 1.5–0.7 MW·h/ton, being significantly lower than that for constrained-impact mills.

The major drawback of this type of equipment is the wearing of grinding parts. Wearing of milling bodies inevitably occurs during grinding; however, the wear of balls in a planetary mill only affects milling efficiency. Meanwhile, wearing of a disintegrator pin may cause its breakage, an avalanche-like impact on many neighboring pins and breakdown of the mill. Therefore, an inspection needs to be carried out for disintegrators and hammer mills, which reduce the daily production capacity of the processing line by 10–20%.

### 2.9. Jet Mills

**Figure 13:**
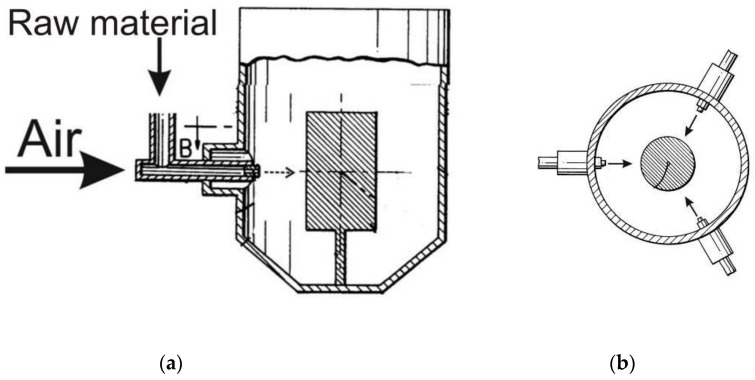
Schematic of a jet mill: (**a**) side view; (**b**) top view. Modified from Smith LS, Mastalski HT. A fluidized bed jet mill, EP Patent 0488637 A2, 1991.

An original grinding approach is embodied by jet mills; instead of moving grinding bodies, the raw material is driven in this grinder (followed by a collision with an immobile body) (Figure 13). Material particles are accelerated to a desired speed in a gas flow. The first jet mills were designed at the turn of the 19th and 20th centuries; however, their use was complicated because of the high cost of equipment for producing compressed gas/air. In the 1990s, gas compression became less expensive, thus giving an incentive for the industrial use of jet mills.

Cyclone mills are a modification of jet mills, where raw material particles collide with each other as they cross each other’s trajectories rather than with an immobile target.

The major advantage of this type of equipment is its low wear. Only the target (which can be manufactured from a durable material) is worn. For cyclone jet mills, there are no wearable parts at all. This advantage allows one to use jet mills in critical fields (e.g., for grinding pharmaceutical ingredients).

Furthermore, compressed air is cooled down as it leaves the outlet nozzle, making it possible to grind even temperature-sensitive raw materials such as enzyme preparations. The size of particles obtained in this type of mill is comparable to that of ball mills and reaches several micrometers.

The main drawback of jet mills is that it is difficult to scale-up the milling process. In order to increase the production capacity of a jet mill, the flow rate and consumption of air need to be significantly increased. Today, the production capacity of commercial jet mills is approximately 10 ton/h. Strict requirements imposed on particle size of the raw material is an important feature of this type of mill. For particles to be carried with the airflow, particles of the initial raw material need to be less than 1 mm in size.

### 2.10. Comparison of Machines of Different Types

Table 1 shows the real examples of the use of various equipment for the purpose of mechanical (size reduction, amorphization) and mechanochemical processing (thermomechanical pretreatment for subsequent heterogeneous hydrolysis, mechanochemical assisted extraction (MAE) of different bioactive compounds).

As shown in Table 1, the literature describes a few cases of processing the same plant materials on different types of mechanochemical equipment. For example, in the work of Podgorbunskikh E.M. it was shown that pure α-cellulose and wheat straw are more efficiently amorphized in the AGO-2 planetary ball mill, but in the activation of α-cellulose, the greatest decrease in the crystallite sizes is observed at shear mode in a flow-through centrifugal roller mill [84]—the specific energy requirement for a centrifugal roller mill is several times lower.

By example of a hardwood and corn stover it is shown that when choosing equipment for grinding plant materials, it is worth considering the nature of the material. Size reduction from 22.4 to 3.2 mm for hardwood specific energy requirement for knife mill is two times lower than for hammer mill, and vice versa for corn stover.

It is also worth noting the typical situation where the authors use term “ball mill” for another type of equipment that uses balls as grinding bodies. For example, the authors of a very useful study comparing the energy consumption of four different methods of processing of plant raw materials use the term “dry ball milling” for the processing of the material in a vibration mill [84]. Therefore, when working with the literature describing the use of mechanochemical equipment, it is important to clarify the type of equipment for specific models given in the methods used.

## 3. Conclusions

Hence, it is reasonable to choose the method for pretreating plant raw material with allowance for the following factors: the optimal combination of the efficiency of the subsequent activation processes, consumption of energy and reagents, as well as the amount of inhibitory agents or side products. Cutting, abrasion, free and constrained impact are useful for plant biomass treatments. Constrained impact or abrasion is useful for composite formation or mechanochemical reactions. Roller and centrifugal roller mills have a high capacity, while being characterized by a lower energy consumption. When only grinding needs to be performed, it is more reasonable to use free-impact mills. The properly chosen type of mechanochemical activation (and equipment) allows energetically and economically sound enhancement of the reactivity of solid-phase polymers by increasing the effective surface area accessible to reagents, reducing the amount of crystalline regions and the diffusion coefficient, disordering the supramolecular structure of the material, and performing mechanochemical reactions with the target substances.

It should be mentioned separately that the overwhelming majority of published studies have been performed using laboratory-scale equipment to obtain fundamental results, as well as to achieve the maximum rates and yields of chemical reactions. Scaling-up to the real-world pilot plant or industrial equipment has a number of challenges requiring additional technological research. Estimation of energy consumption for grinding and mechanical activation infers that the economic effectiveness of integrated processes involving these manufacturing procedures largely depends on energy consumption during the mechanical pretreatment stage.

## Figures and Tables

**Table 1:** The examples of the use of various equipment for the purpose of mechanical (size reduction, amorphization) and mechanochemical processing.

Material	Equipment	Effect	Specific Energy Requirement, kWht^−1^	Ref
Purpose: Size Reduction and/or Amorphization				
Hardwood, 6% wet	Disc Mills	Size reduction from 19.05 to 1.6 mm^−1^	200–400	[80]
Hardwood, 11% wet	Vibration mill	from 22 to 0.15 mm	800	[81]
Hardwood, 4–7% wet	Hammer mill	from 22.4 to 1.6 mm	130	[82]
		from 22.4 to 2.5 mm	120	
		from 22.4 3.2 mm	115	
		from 22.4 to 6.35 mm	95	
	Knife mill	from 22.4 to 1.6 mm	130	
		from 22.4 to 2.5 mm	80	
		from 22.4 3.2 mm	50	
		from 22.4 to 6.35 mm	25	
		from 22.4 to 9.5 mm	15	
		from 22.4 to 12.7 mm	8	
Corn stover, 6.2% wet	Hammer mill	from 7.15 to 0.8 mm	22.1	[83]
		from 7.15 to 1.6 mm	14.8	
Corn stover, 12% wet		from 7.15 to 0.8 mm	34.3	
		from 7.15 to 1.6 mm	19.9	
Corn stover, 4–7% wet	Hammer mill	from 22.4 to 1.6 mm	14.0	[82]
		from 22.4 to 3.2 mm	6	
	Knife mill	from 22.4 to 3.2 mm	20.0	
		from 22.4 to 6.3 mm	15.0	
		from 22.4 to 9.5 mm	3.2	
		from 19.05 to 3.0 mm	12.0	[80]
Wheat straw, 4–7% wet	Hammer mill	from 19.05 to 1.6 mm	42	[82]
		from 19.05 to 2.5 mm	29	
		from 19.05 to 3.2 mm	21	
	Knife mill	from 22.4 to 1.6 mm	7.5	
		from 22.4 to 2.5 mm	6.4	
		from 22.4 to 6.3 mm	5.5	
Wheat straw, 5.4% wet	Planetary ball mill, 2 min	Size reduction from 0.24 mm to 0.19 mm, Crystallinity index, % (CrI, %) from 82 ± 2 to 78 ± 2	About 150 *	[84]
	Planetary ball mill, 10 min	Size reduction from 0.24 mm to 0.015 mm, CrI, % reduction from 82 ± 2 to 39 ± 5	About 750 *	
	Centrifugal Roller Mill, activation mode 600 rpm	Size reduction from 0.24 mm to 0.17 mm, CrI, % reduction from 82 ± 2 to 64 ± 2	23	
	Centrifugal Roller Mill, activation mode 1200 rpm	Size reduction from 0.24 mm to 0.038 mm, CrI, % reduction from 82 ± 2 to 65 ± 5 %	87	
**Purpose: Thermomechanical Pretreatment for Subsequent Heterogeneous Hydrolysis**				
Lignin-rich plant biomass (reed biomass)	Attritor, 10 ℃, 20 min, 600 rpm	The yield of enzymatic hydrolysis increasing from 13.1 ± 0.3 to 20.8 ± 0.4, Size reduction from 1 mm to 0.070 ± 0.004 mm CrI, % reduction from 69 ± 6 to 42 ± 5	-	[85,86]
	Attritor, 180 ℃, 20 min, 600 rpm	The yield of enzymatic hydrolysis increasing from 13.1 ± 0.3 to 14.8 ± 0.4, Size reduction from 1 mm to 0.075 ± 0.005 mm CrI, % reduction from 69 ± 6 to 59 ± 5. Molten lignin leaves the cell wall structure, forming pores, and is accumulated on the surface of the cell walls, preventing enzymatic conversion	-	
Rice straw	Wet disc milling, 1 round	Sugar yields, % increasing from 22.5 ± 1.3 to 39.3 ± 0.1 CrI, % reduction from 51.9 to 46.9	83.3	[87]
	Wet disc milling, 3 round	Sugar yields, % increasing from 22.5 ± 1.3 to 43.1 ± 5.2 CrI, % reduction from 51.9 to 50.8	250	
	Wet disc milling, 5 round	Sugar yields, % increasing from 22.5 ± 1.3 to 51.3 ± 5.3 CrI, % reduction from 51.9 to 46.0	639	
	Wet disc milling, 10 round	Sugar yields, % increasing from 22.5 ± 1.3 to 67.5 ± 5.1 CrI, % reduction from 51.9 to 48.6	1500	
	Vibration mill, 1700 rpm, 5 min	Sugar yields, % increasing from 22.5 ± 1.3 to 67.5 ± 5.1 CrI, % reduction from 51.9 to 46.7	2500	
	Vibration mill, 1700 rpm, 15 min	Sugar yields, % increasing from 22.5 ± 1.3 to 67.5 ± 5.1 CrI, % reduction from 51.9 to 35.0	7500	
	Vibration mill, 1700 rpm, 30 min	Sugar yields, % increasing from 22.5 ± 1.3 to 67.5 ± 5.1 CrI, % reduction from 51.9 to 25.2	15,000	
	Vibration mill, 1700 rpm, 60 min	Sugar yields, % increasing from 22.5 ± 1.3 to 67.5 ± 5.1 CrI, % reduction from 51.9 to 13.3	30,000	
Bagasse sugarcane	Vibration mill, 1 h	glucose yields of 69.8 %, CrI, % reduction from 64.5 to 28.9	11,720	[88]
	Vibration mill, 3 h	glucose yields of 95.2%, CrI, % reduction from 64.5 to 2.5	25,340	
	Ball mill, 24 h	glucose yields of 55.2%, CrI, % reduction from 64.5 to 53.3	19,540	
	Ball mill, 72 h	glucose yields of 75.2%, CrI, % reduction from 64.5 to 38.4	53,650	
	Centrifugal mill screen size of 0.12 0.5 mm	glucose yields of 38.2%, CrI, % reduction from 64.5 to 60.8	1050	
	Centrifugal mill, screen size of 0.12 mm	glucose yields of 53.9%, CrI, % reduction from 64.5 to 48.5	4980	
	Jet mill, 4000 rpm, 15 min	glucose yields of 60.0%, CrI, % reduction from 64.5 to 58.1	66,780	
	Jet mill, 5000 rpm, 15 min	glucose yields of 61%, CrI, % reduction from 64.5 to 53.5	66,850	
	Twin-screw extruder	straw hydrolysis yield of 68.2%, CrI reduction from 57.3 ± 1.25% to 54.0 ± 0.23%	5600–8500	[89]
	Twin-screw extruder, alkali-combine pretreatment combined with ionic liquids	Glucose yields of 90%	-	[90,91,92]
**Mechanochemical Assisted Extraction (MAE) of Different Bioactive Compounds**				
Bay leaves (Laurus nobilis L.)	Planetary ball mill, 400 rpm, 10 min	The total extraction time—an reduction of more than 10-fold	-	[93]
Platycodon grandiflorum	Planetary ball mill	Extraction time and temperature reduction, the yield of platycodin D increasing (7.16 ± 0.14 compared to 4.18 ± 0.11 mg/g), water instead of organic solvent	-	[2]

* Calculated by the authors of the review based on the average consumption of the mill for a load of 20 g.
